# O30 Case report of Cogan syndrome in a child with audiovestibular dysfunction and ocular inflammation

**DOI:** 10.1093/rap/rkab067.029

**Published:** 2021-10-19

**Authors:** Samy Zakout, Shwan Mohamad

**Affiliations:** 1 Medcare Hospital, Sharjah, UAE; 2 British Society of Rheumatology, London, United Kingdom; 3 Royal College of Physicians, London, United Kingdom; 4 Al Zahra Hospital, Sharjah, UAE

## Abstract

**Case report - Introduction:**

Cogan syndrome is a very rare disorder of probable autoimmune vasculitic origin, first described in 1934. The age of onset seems to range from 3 to 50 years with an average age of disease onset at 29 years. It is characterised by audiovestibular dysfunction and ocular inflammation. The cause remains unknown and the epidemiology of the disease is purely based on case reporting. As of 2015, there were fewer than 250 case reports. Interestingly, it seems extremely rare in Arabic and Middle Eastern countries in which a new case has been recently diagnosed and is being reported in this abstract.

**Case report - Case description:**

A 14-year-old girl of Arabic origin first presented to the ENT specialist with a 3-week history of balance problems, left tinnitus and sensorineural hearing loss in both ears worse on the left. She first received treatment for vestibular neuritis with no improvement. Two months later, she complained of bilateral red eyes and excessive lacrimation when diffuse interstitial keratitis was diagnosed by the ophthalmologist. An array of blood tests including a full autoimmune disease was arranged and a rheumatology opinion was sought.

The patient had a normal inflammatory response and her screen for antinuclear antibodies, extractable nuclear antigen, rheumatoid factor and antineutrophil cytoplasmic antibodies were all negative. A full virology screen was also negative. Having failed to respond to all supportive treatment modalities, a trial of a tapering 2-week course of prednisolone starting at 25mg daily was instigated. The patient’s symptoms improved by more than 50% on glucocorticoids subsequent to which the diagnosis of Cogan syndrome was anticipated. As the patient’s symptoms relapsed when glucocorticoids were stopped, she was recommenced on prednisolone 25mg in line with azathioprine 100mg daily. The patient’s hearing, which was almost lost in the left ear, improved significantly by more than 70% initially, and then to normal hearing as stated by the patient supported by special ENT testing. Her balance problems and vertigo also improved remarkably. Her tinnitus, which was the last symptom to resolve, almost went away over the subsequent 2 months. The patient had already come off glucocorticoids 6 months down the line, and stayed on azathioprine 75-100mg daily for almost a year after which gradual weaning was trialled and then eventually succeeded. She has now been off azathioprine for 6 months without experiencing any disease relapse.

**Case report - Discussion:**

Cogan syndrome can be difficult to diagnose particularly in children. The diagnosis is essentially clinical depending on the presence of audio-vestibular symptoms and interstitial keratitis along with a prompt response to immunosuppressive medication.

In this patient, the consistent clinical picture supported by the positive response to glucocorticoids especially in the absence of an alternative diagnosis would have made the diagnosis of Cogan syndrome likely.

Given the rarity of this disorder, there are no classification criteria as yet hence the ongoing need for case reporting. Moreover, there is no clear guidance on the duration of immunosuppressive treatment of this condition.

**Case report - Key learning points:**

It is pivotal in managing conditions with a suspected autoimmune origin to have a multidisciplinary approach involving other relevant specialties in a holistic approach.

The prompt response to glucocorticoid therapy still remains the key in securing the diagnosis of a number of conditions in clinical practice especially in the absence of diagnostic criteria and/ or supportive investigations such as serological and tissue-based testing.

Case reporting has a crucial role in enhancing awareness of rare medical conditions.

